# Britain and Europe’s Gifted Children in the Quests for Democracy, Welfare and Productivity, 1970–1990

**DOI:** 10.1017/S0960777322000078

**Published:** 2022-11-15

**Authors:** Jennifer Crane

**Affiliations:** School of Geographical Sciences, University of Bristol, Bristol, United Kingdom

## Abstract

Policy, voluntary, psychological and educational interest in gifted children emerged across Europe in the early twentieth century but surged dramatically in the 1970s and 1980s. This article explores the transnational voluntary circles hoping that gifted youth would bring peace and liberal democracy across Europe in these years. It analyses, also, how such work came into conflict with the expectations of conservative press in Britain: that gifted children would in fact bolster national economic progress. Critically, the article demonstrates that parents and children, drawing on professional and cultural capital, resisted ideas of gifted youth as global assets. Interest in giftedness revealed the growing ‘agency’ of articulate, affluent, middle-class families within the contexts of individualism and neoliberalism, but also its limits. Further, we can and must centre the experiences of young people in our scholarship, to truly understand how ‘agency’ operates within families and beyond.

In September 1977, one mother wrote to the *Daily Telegraph* to bemoan a difference of opinion with her child’s teachers: while the mother and her partner felt that their child was ‘gifted’, and should be transferred a year early from infant to junior school, teachers had not recommended this.^1^ The school’s decision had been supported by officials from the local education department, the Department of Education and voluntary groups the Advisory Centre for Education and the Society for Gifted Children.^2^ For the parents, this case had, the mother wrote, shattered their view of Britain as a ‘democratic society’.^3^ Indeed, she continued, the education system was a ‘bureaucratic dictatorship’, ‘however benign’, closing her letter with: ‘We refuse to accept that any of our children should ever belong to the State’.^4^ These powerful and dramatic words were, of course, a highly positioned source written by one disgruntled parent. Reflecting on this, the mother in fact described herself and her partner as ‘self-appointed members of the articulate middle-class’.^5^ Nonetheless, this letter reflects an important strand of thinking emergent in British conservative politics of the 1970s and 1980s, expressing anxieties about the interventions and role of the welfare state and particularly a crisis of confidence in its educational systems. These anxieties foregrounded the three electoral victories of Conservative Prime Minister Margaret Thatcher in 1979, 1983 and 1987, and a political shift towards economic liberalism, individualism and social conservativism.

This article uses the case study of so-called ‘gifted’ children to analyse changing ideas of family autonomy and democratic participation, voluntary cultures and ‘appropriate’ levels of state welfare and intervention; debates which raged in Britain and Europe in the 1970s and 1980s. Policy, voluntary, psychological and educational interest in gifted children emerged across Europe in the early twentieth century but surged dramatically in these decades. Definitions of ‘giftedness’ varied dramatically, but by the late twentieth century giftedness was generally assessed through standardised intelligence, or ‘IQ’, testing, with children identified as ‘gifted’ if they scored 130–140 or higher – around 1 to 2 per cent of the child population. Children labelled as ‘gifted’ were hence, by definition, a very small group. Furthermore, the British state never rolled out universal intelligence testing, meaning that even fewer children were labelled – typically only those whose schools, psychologists or parents sought out such assessment. By the 1980s and particularly in the 1990s, research in educational sociology and psychology demonstrated that these biases – as well as issues with *who* constructed intelligence tests, and which assumptions were embedded within ‘neutral’ questions – meant that white, middle-class boys were disproportionately likely to be identified as gifted.^6^

Recognising the inherent and historical biases structured within this term, this case study is nonetheless important because the *idea* of the gifted child – its potential and promise – carried significant weight in British and European policy, professional circles and culture of the late twentieth century. Ideas about giftedness also reshaped daily life, and enabled and motivated new forms of activism from children and parents – again typically white, middle-class children and parents – alike. This article traces these changes. First, the article explores the transnational circles of researchers and voluntary campaigners hoping that gifted youth would bring peace and liberal democracy across Europe, guiding the continent through the decline of communism. Second, the article analyses a contrasting expectation from conservative press and policy in Britain: that gifted children would in fact bolster national economic progress, overcoming crises of productivity and industrial decline. Third, the article explores how both of these visions conceptualised *parents* (not whole families) as responsible for identifying their gifted offspring, and for developing them into these transnational or national assets. This vision was replicated in advertising, commercial culture, educational manuals, television and popular press. The fourth and fifth sections of this article assess how and when articulate parents and children themselves, drawing on their professional and cultural capital, and the growing power of experiential and emotional expertise, were able to renegotiate and resist these ideas.

As Siân Pooley, Kaveri Qureshi, Adrian Bingham and Angela Davis have argued, ‘the state’, newspapers, psychology and popular culture do not have sole authority over how people have interpreted their own lives.^7^ While it is difficult to get past ‘expert’ representations of parent and child voice, the article argues that affluent and eloquent middle-class parents were empowered within new voluntary organisations in this area – notably the National Association for Gifted Children, established in 1966 – and able to use these platforms to garner media interest, and to direct it to the challenges of raising a gifted child.^8^

Children likewise used the spaces available to them – such as new child-centred magazines produced by the National Association – to playfully describe their own experiences of growing up gifted, and to criticise their schooling and perceived stigma and discrimination from peers. As such, parents and children, while working separately, both worked within new systems of support to challenge emphasis on the *future* role of the gifted child, and instead to push debate towards lived experience in the present. Notably, looking at these groups separately in the article shows that parents used their platforms to seek out solidarity, sympathy and new communities, while their children demanded national and international change: we cannot assume that ‘families’ ever acted as coherent singular units.

The methodology of this article follows the influential work of Elaine Tyler May in representing large-scale transnational political cultures and intimate and emotional family lives as ‘two sides of the same coin’.^9^ This is achieved by placing sources directed at and written by educational, policy, media and psychological ‘experts’ alongside those constructed by families. This article also takes statements made by contemporary parents and children seriously, even if disseminated through partnership with media and the voluntary sector; it assumes that families exercised ‘agency’ in these partnerships. The article does not merely ‘uncover’ the agency of parents and children as an ‘endpoint’ of analysis.^10^ Rather, drawing on new theoretical scholarship, it explores the specific nature of the agency exercised by these families.^11^ Parents of gifted children held multiple forms of experiential, cultural and professional expertise, and were often sophisticated users of new educational, media and psychological discourses around giftedness. Yet, these parents also, in numerous emotional accounts written to one another and to voluntary groups, as well as for public view, chose to describe the significant daily challenges of raising children they did not always understand. Their voluntary action was then perhaps driven in part by a sense of entitlement as middle-class citizens of a welfare state, but it was articulated in terms of experience and emotion. The children involved, likewise, were privileged in terms of being labelled as ‘gifted’; voluntary and policy actors were particularly interested in hearing from children categorised in this manner. Yet, again, many children’s testimonies emphasised that this label brought burdens, as well as benefits, and that children labelled as ‘gifted’ felt isolated from peers and families, and that they faced higher expectations in their immediate and global communities. In this period, then, parents and children alike believed that articulating emotion could be highly powerful.

Overall, this article argues that contemporary anxieties about the changing role of the British state – as a peaceful, productive or universalist nation; as standing alone, within Europe or as a ‘world-leader’ – mounted social pressures on parents to identify their gifted youth and to mobilise them for national benefit. Parents, in response, utilised this interest to challenge their children’s role in national futures, and instead to discuss familial and child ‘happiness’ and ‘wellbeing’. Parents shared their emotions with one another privately and in public, finding articulate representatives to capitalise on a moment where educational and psychological systems, and professional expertise more broadly, were under attack. In doing so, families working from the 1970s were a key driver in facilitating ideological shifts towards ‘popular individualism’, which drove the later electoral success of Thatcherite politics.^12^ Yet, as Jon Lawrence has argued, this familial individualism was not necessarily ‘selfish’ or isolated, but rather represented shifting visions of citizenship and community: for these families, local, national and transnational communities of support were formed through new voluntary and media spaces, even as families critiqued the traditional support mechanisms of the state.^13^ Families, then, were not always vulnerable or broken in the 1970s and 1980s, as facets of political debate, psychiatry and popular culture emphasised.^14^ Gifted families were often ‘strong’, acting as critical leaders in the flourishing of a diverse new range of voluntary cultures. Families constructed influential localised spaces within which to resist and reshape national quests for welfare, democracy and productivity – we cannot understand the ideological or lived significance of these concepts without exploring family life.

## Agents of a United Europe?

The earliest interest in formalised intelligence testing developed in Europe at the turn of the twentieth century. In the early 1900s, the French Ministry of Public Instruction commissioned Alfred Binet to produce an intelligence test, initially to identify children who were struggling within mainstream education; a project he worked on with Theodore Simon.^15^ Scales of ‘IQ’ were developed in Germany in the 1910s by Wilhelm Stern.^16^ Debates in this moment were inflected by visions of family life: the 1905 test packets for Binet’s intelligence testing included a vision of ‘normal’ childhood, whereby a mother was presented in a kitchen with her three children and a family pet.^17^ Thus, even from its inception as a tool to identify *less able* children, intelligence testing was quickly tied to visions of intellectual potential, national progress and to the idea that families – constructed in a gendered and hierarchical manner – would play a key role in ensuring that children met their ‘potential’. These debates were shaped by contemporary psychological, policy and popular interest in eugenics, demographic decline and ‘national stock’. Mathew Thomson has demonstrated that these anxieties were further strengthened in the interwar period, as mental hygiene movements sought to connect wartime animosity to ‘a European mental malaise’.^18^ Giftedness was most closely tied to nationalist and eugenicist thought by 1930s and 1940s National Socialist ideology which, Julia Barbara Köhne has argued, used ideas of ‘genius’ to underlie charismatic leadership and systems of oppression and mass genocide.^19^ This extremist ideology demonstrated the potential dangers of delineating ‘the gifted’ in society, and, as a consequence, interest in giftedness declined significantly across Europe in the immediate post-war years.

Nonetheless, by the 1970s and particularly in the 1980s, groups of voluntary and academic leaders reignited European interest in giftedness. In these new transnational debates, gifted children were potential agents through which to promote unity, peace and co-operation across Europe – an interest which peaked in the late 1980s and early 1990s amidst the 1989 revolutions and fall of communism in Eastern and Central Europe. Indeed, in 1991, the Council for Cultural Cooperation of the Council of Europe brought together researchers, voluntary leaders and policy makers who had been working in this area for several years at a conference held in Nijmegen, the Netherlands. Contributors hailed from Sweden, the United Kingdom, Germany, Switzerland, Poland, the Netherlands, Turkey, Azerbaijan, Italy, Spain, Czechoslovakia, Romania, Austria, Denmark, Yugoslavia, France and Finland.^20^ Many debates at this conference looked to understand national differences, for example in terms of how gifted children were identified and treated. Discussants found no simple correlation between liberal democratic governance and the presence or ‘success’ of gifted education. Indeed, contributors found an ‘almost complete absence’ of research about giftedness in France and Switzerland, as well as arguing that research in this area had been hindered in Poland by political ideology, economic conditions and social attitudes.^21^

While national distinctions were recognised and probed, much of this workshop centred on the importance of ‘East-West cooperation’.^22^ Such co-operation was to serve two purposes. First, co-operation would enable all nations to better identify, manage and develop gifted youth. Conference participants emphasised the need to link their nations through culture-comparative longitudinal studies, the creation of resources in multiple European languages and transnational funding and evaluation programmes.^23^ Second, however, gifted children were to be active contributors to, as well as beneficiaries of, a united Europe. Writing from Germany, itself going through a process of reunification, Kurt A. Heller, a psychology academic, wrote that:

The opportunities of a united Germany and its integration into the European and non-European international community will only be successful if the mental resources of our young people can be successfully motivated.^24^

In this formulation, gifted children must use their ‘mental resources’ to heal the divisions of the Cold War – whether between or across countries – acting as ‘agents of future promise’.^25^ This rhetoric – which Heller argued was ‘gaining world-wide acceptance’ – was echoed across debates from this conference and also in publications of the European Council of High Ability. In 1994, for example, this organisation’s newsletter emphasised that members were ‘working together for the sake of a better world and our common future’.^26^ In this formulation, gifted children had a responsibility to their nation-state, but also to broader global formations across Europe and the world.

These visions of the gifted as transnational agents had real effect on the daily lives of children. As we will see, voluntary and policy rhetoric changed children’s education, their commercial environments and their family lives. Notably, the European quest for democracy was translated by voluntary organisations into international exchange schemes, enabling gifted children to explore, and to build connections, across Europe. One such exchange was documented in a 1991 newsletter from Britain’s National Association for Gifted Children. Writing to this organisation, a child reported that they had been sent to Hungary. The newsletter explained that this child was a ‘pioneer candidate’ for a broader scheme of exchanges, designed to satisfy a Hungarian ‘longing to be acknowledged by the West and to make friends’. While not discussing the mechanics or funding of this scheme, the child expressed surprise that the country ‘wasn’t that far away’, and that their hosts had had a Nissan car.^27^ A later exchange scheme in 1994 saw thirty-eight British and Hungarian children enjoy ‘an East European adventure’, including visits to the residential centre at Lakitelek, ‘birthplace of the recent revolution against communism’, and sponsored by the British Embassy in Budapest and the Foreign Office, among others.^28^ These schemes have not left substantial archival traces: they were patchwork and likely involved very small numbers of children. Yet, they operate within a broader history of ‘child diplomacy’, whereby, as Matthias Neumann has demonstrated, liberal democratic and authoritarian states alike expected young people to foster international relations during the Cold War.^29^ As Richard Ivan Jobs has further shown, young adults themselves simultaneously often relished the opportunities to act as ‘Backpack Ambassadors’, travelling Europe and exchanging cultural views.^30^ Indeed, the limited traces available of these distinct schemes for gifted children reveal the ways in which children’s life experiences were reshaped by academic and voluntary ideas about democracy and European unity. In the above child’s case, their journey to Hungary reshaped their understanding of the very spaces, distances and geography of Europe, and also of industry, commerce and daily life across the Iron Curtain. This case study leaves unanswered, however, how the gifted young were received on these exchanges, and if they did act as diplomats to represent a distinct vision of ‘British’ or ‘European’ values.

## Fuelling British Economic Recovery?

Closely focused debates between academics, researchers and voluntary leaders thus sought to situate the gifted child as a key player in forging transnational trajectories towards democracy and peace across Europe. This article now turns to a vision of gifted children explored by British conservative commentators in the same decades, the 1970s and 1980s. Often led by conservative tabloid press anxious about Britain’s membership of the European Economic Community, these debates rejected ideas of the child as a transnational democratic agent.^31^ Instead, in this vision, gifted children were reconfigured as economic agents, able to improve *national* economic performance and to reverse perceived decline. This thinking has significant historical precedent: in conservative thought, families have long been seen as a useful driver of economic growth, keen to invest in their children’s advancement.^32^ Yet, the specific press representation of gifted children in the 1970s and 1980s was also deeply of its time. In this case, resurgent popular conservative press used new interest in the gifted child to critique a range of perceived ills distinct to the mid-to-late twentieth century: the post-war welfare settlement, 1960s equalities legislation and imperial decline.^33^ The proposed solution of these newspapers – reversing these ills – was framed in terms of contemporary interest in human capital, often drawing on a perceived association between the gifted child and scientific and technological capacity.^34^

The *Daily Mail* – one of the ‘most popular national morning newspapers’ of the twentieth century, with an early interest in cataloguing ‘private life’– regularly sought to link interest in gifted children to concerns about national decline, ‘wasted’ resources and the loss of ‘our future’.^35^ Articles about gifted children frequently featured in the paper’s sections on education, the letters pages and in ‘Femail’– a lifestyle segment directed at women. In April 1979, one letter-writer emphasised that ‘the brains’ of ‘young people’ were the ‘most important asset that Britain possesses’– language repeated in an article of August 1979 bemoaning the ‘extraordinary neglect of our greatest hidden asset – The Gifted Child’.^36^ In further *Mail* coverage, gifted children were presented as key to enabling Britain’s ‘future prosperity and security’ and as ‘the guardians of Britain’s future’.^37^ These articles proceeded to argue that this ‘asset’ and, hence, Britain’s future, was being squandered because of its contemporary concerns: an ‘equality-obsessed society’, ‘geared to the ideal of equality at all costs’, which as a consequence did not sufficiently support or separate out gifted youth.^38^ While most prominent in *Mail* coverage, this vision of the gifted child as an economic and at times a moral asset was also represented in other conservative tabloids, for example in the *Daily Express*’s warning of 1973 that Britain was ‘not so rich in natural resources that it can afford to let slip its greatest wealth – brainpower’.^39^

These visions were also framed by anxieties about imperial decline and the changing position of Britain in the world. Conservative tabloid press and parliamentarians contrasted Britain’s ‘waste’ of gifted children to their perceived utility within, in particular, the Soviet Union. In a House of Lords debate of 1969, conservative peer Lord Carrington emphasised that, ‘I believe that in Russia if a child is particularly gifted in mathematics, for example, he is sent to a school which specialises in that subject’.^40^ In Britain, by contrast, Carrington suggested that the ‘nation’ was neglecting ‘those who are going to make the greatest contribution to our society, whether it be in the field of arts, science or technology’.^41^ This idea was frequently reiterated in the tabloid press: in 1973, the *Daily Express* quoted a retired teacher who stated that, ‘In Russia, they segregate many of their exceptional children into special schools’.^42^ In 1979, in the *Daily Mail*, June Southworth argued that in Russia gifted children would be recruited at fifteen by ‘academic Olympiads to university boarding schools in four major cities’.^43^

When the potential of gifted children was at stake, then, factions of conservative tabloid press and parliamentarians expressed great interest in the Soviet system of education, stating that this system, while authoritarian, enabled ‘men of strong will’ to act decisively, and to quickly establish specialist schools.^44^ Such admiring comparisons were not isolated to debates around giftedness alone, and had precedents in preceding decades. While showing that civil servants remained sceptical, Glen O’Hara has argued that political speeches and reports of the 1950s and 1960s praised the ‘formidable Soviet challenge in the education of scientists and technologists’.^45^ Looking at media and parliamentary voices in 1970s and 1980s Britain makes clear that this strand of thinking remained visible in cultural life.

Hence, thinking about giftedness contributed to a process in which leaders and journalists in Britain rethought their political systems, but also simultaneously reflected on whether the problems of the gifted, beyond Russia and more broadly across East-West borders, were ‘much the same as ours’.^46^ Interest in comparing national systems was also present within the research work of the National Association for Gifted Children. Its leader of 1977, Henry Collis, a former teacher, reflected after a visit that Bulgaria’s leading Communist Party had, through central planning, been able to develop a ‘far-reaching plan’ for identifying and nurturing gifted youth, and was also planning logistical and financial support for voluntary action and residential courses in this area.^47^ This comparative focus was echoed in 1991 – amidst the collapse of the Soviet Union – by sportsman Sebastian Coe, who was to become a Conservative Member of Parliament the following year. Writing for the *European Journal of High Ability*, Coe argued that looking at sporting ability across Europe demonstrated that ‘many forms’ of cultural and political system could foster ‘talent’: ‘whether Capitalist or Communist, the latter clearly shown in East German sporting success or the Russian domination in chess’.^48^ While emphasising that ‘freedom’ *could* be crucial for developing excellence, Coe also emphasised that high achievers may ‘mirror a way of life’ and be ‘powerful advertisements for their own culture and products’.^49^

Thus, 1970s and 1980s debates in research, policy and voluntary circles offered a vision of the gifted child as an agent of democracy and peace across Europe. Voluntary experts and researchers from Britain played a significant role in these debates – Dr Joan Freeman, a British psychologist, was an initial founder of the European Council for High Ability, for example, and Britain’s National Association for Gifted Children were the first organisers of the World Conference on Gifted Children, held in London in 1975.^50^ This vision nonetheless contrasted dramatically with a concurrent vision most prominently offered by British conservative press: the vision of gifted children as economic assets, central to fuelling national productivity – an issue keenly felt amidst the decline of empire and economic crisis. In this formulation, discussions of European partners were couched not in terms of stabilising a new utopian system of liberal democracy, overcoming historic divides fraught by communism, fascism and world wars. Rather, comparisons with Europe were framed around economic trade, or around learning from how other countries – and particularly those to the east of Europe – were able to mobilise centralised systems to identify and manage the gifted.

The case study of giftedness thus showcases two powerful visions of Europe present in the closing decades of the twentieth century. First, a more porous and collaborative Europe, with new voluntary and research formations collectively looking at giftedness and aiming to use research in this area to spread liberal democratic thought from West to East. A second vision which emerged was a fractured Europe framed by economic and social competition, where Eastern Europe offered pertinent solutions around efficiency and management. In part, this suggests the ways in which liberal democratic welfare systems, such as Britain, at times considered limiting their social obligations to certain groups of children. Media and voluntary critics alike questioned liberal and voluntary traditions in Britain and Europe, because of a perceived neglect of the needs of the gifted. Giftedness became a flashpoint for revealing the limits of the social democratic post-war welfare settlement and for demonstrating how it was carefully bounded by anxieties about productivity, human capital and reciprocal duties held by citizens. In this context, we see how the destabilisation of East-West divides across Europe in the 1970s and particularly in the 1980s had widespread impacts across such diffuse debates as those in education and child development. This article continues to consider how and when these dissonant sets of expectations were communicated to, and resisted by, families, and it argues that families themselves displayed significant agency in making giftedness a political and voluntary concern.

## The Parents’ Responsibility?

In Britain, responsibility for identifying and developing the gifted child – whether as the European agent of democracy or the productive British economic asset – was placed firmly onto parents by tabloid newspapers, television, voluntary groups and advertising. Newspapers from the 1970s to the 1990s offered multiple headlines demanding to know, ‘Is there a genius in your family?’, ‘How to spot a gifted child’, and reporting on psychological research about ‘how to discover if there is a gifted child in your family’.^51^ Once parents had identified their gifted child, newspapers emphasised, they should make sacrifices to ensure that they made ‘progress’ and attained educational ‘success’. Framing parental sacrifice as a common feature of family life, reports from the National Association for Gifted Children referenced families who had sold their houses and made significant financial changes to ‘raise the cash’ so that their child could attend private school.^52^ In addition to financial work, television documentaries also encouraged parents to utilise their time wisely to ensure that their children continued to exceed developmental markers. Parents were advised to: read more to their potentially gifted youth, talk to them ‘a lot’, respond to their interests, discuss their school with them and consider approaching voluntary groups to provide ‘extra stimulation’.^53^ Parents were thus expected to develop enhanced skills of educational psychology, teaching, social work and care.

The use of giftedness in advertising likewise demonstrates that businesses sought to draw on, utilise and further construct an expectation that all parents must ‘develop’ and ‘realise the full potential of’ their children, amidst thriving consumer culture.^54^ As early as 1950, before policy and voluntary interest in giftedness surged, an advertisement for the malted hot drink Horlicks, placed in the conservative, middle-class newspapers *The Times* and *The Daily Telegraph*, advised that the parents of gifted children must ‘know real pride’ in their ‘child’s achievements’ and ‘new success’.^55^ However, with pride came responsibility: these relatively affluent parent-readers *must*, the advert suggested, also ‘guard those gifts’ as parents ‘have a part to play in forming your child’s future’.^56^ Horlicks, of course, could provide the child with necessary ‘*extra* nourishment’ for the children’s distinct psychological and physical needs.^57^ While visions of the future were hence significant in this advert, emphasis was placed on the future of the child rather than the future of the British economy or united Europe – debates which came to the fore two decades later. Nonetheless, this advert provided an early glimpse into the developing assumption that parents, rather than the welfare state, held the critical duties to foster the development of children with ‘potential’. Notably, in media and commercial representations the construction of the parent was not gendered here – the parent invoked was a universal figure, solely constructed to support the child. Nonetheless, when this figure was gendered in later works, emphasis was typically placed on the mother – an assumption which lingered particularly in literature from child development and psychology.^58^ The ways in which the tabloid press especially addressed mothers and fathers in this period, when discussing giftedness, were suggestive of a recognition that all parents were interested in reading about and identifying this phenomenon – and, as we will see, parental interests drove media analysis as much as they responded to it.

This vision had powerfully taken hold by the early 1990s and was visible in the market of vitamins designed to enhance IQ, such as Vitachieve, Tandem IQ and Boost IQ.^59^ Advertising for these products sought to target a broad range of parents, buying space in a range of conservative and left-wing, tabloid and broadsheet newspapers, including the *Daily Mail, The Times, Guardian* and *Daily Express*. Nonetheless, the idea that children’s ‘natural potential’ must be exploited remained key to these adverts, as did the assumption that this work was the responsibility of parents.^60^ In this context, the purported discovery of such vitamins was described as: ‘Extraordinary News for Parents’ and commercial advertising in fact an ‘Important Notice to All Parents’.^61^ While a scientific discovery, and advertisements praised the significance of scientific ‘experts’, parental authority was simultaneously bolstered by the emphasis that: ‘Only parents can make the decision about whether their child should have a vitamin supplement’.^62^

Thus, through media, policy, voluntary and commercial representations, responsibility for identifying and nurturing the gifted child – key in national and international policy rhetoric of the 1970s and 1980s – was pushed onto parents specifically. This focus on parents reflected contemporary professional interest in the significance of the home environment as a space of great potential, or indeed also of great danger, to children.^63^ Interest in empowering parents also, conversely, reflected growing cultural critique of the role of ‘experts’, and new assertions about the value of familial and experiential expertise.^64^ At the same time, families were living increasingly independently from local communities and extended families, in smaller and more focused units.^65^ Together, these contexts led media, policy, voluntary and commercial agents to target the family as a space through which to realise the national and international potential of gifted children. This article will argue that these representations also represented growing parental demands for visibility and provided parents with spaces for activism and agency.

But *which* parents were the focus of commercial and media analysis, and indeed which parents were able to exploit the voluntary opportunities this article will proceed to explore? While advertising sought to target as many parents as possible, the debates of policy, voluntary sector and the media more broadly often contained ingrained assumptions that gifted children were more likely to live and thrive within affluent families. This assumption was visible in a House of Lords debate of 1969, one of the earliest parliamentary interventions in this area, where peers expressed concern that ‘less well-off sections of the community’ may not be able to recognise that their child was gifted.^66^ To alleviate this, hereditary peer and Conservative Member of Parliament Lord Aberdare suggested that society could not necessarily ‘rely’ on all parents, but rather that health visitors or midwives should look to identify gifted children or even especially ‘alert babies’.^67^ While affluent parents were expected to identify their gifted youth, and to appropriately raise them to meet their potential, the welfare state might have to intervene within socioeconomically disadvantaged families. Concerns about the fate of the gifted child ‘from a culturally impoverished home’, thus facing an ‘an everincreasing handicap’, were echoed in this debate by Lord James of Rusholme, a former teacher who had been made a life peer in 1959, who believed that elite education should be provided to *all* intelligent children, regardless of background.^68^ Hence, giftedness was tied to cultural and economic markers of affluence in debate, and peers expressed anxieties about possibly missing gifted youth from certain groups.

These assumption were furthered by media, for example in contemporary television programming which juxtaposed discussions of a parent ‘capable of understanding her [child’s] intellectual needs’ with visions of smartly-dressed adults wearing suits, necklaces and visible markers of middle-class respectability.^69^ Conservative tabloid newspapers also took this association into the terrain of genetics, suggesting that gifted children likely had their genes ‘in the right place’.^70^ Again, while emphasising that ‘genius’ could ‘crash into the most humble home’, tabloids also frequently expressed surprise and fascination, and dedicated lengthy profiles, when working-class families did produce gifted off-spring.^71^ In July 1980, for example, a *Daily Mail* article profiled a forthcoming captain of Eton College, who attended this school with a ‘working class’ background: the paper avidly reported that his grandfather was a printer for the *Daily Mirror*; his father sold car spaces; his mother was a bank clerk and a sister worked for the local vet.^72^ Ideas of whiteness also typically framed media discussions and representations of gifted youth, although British newspapers also paid attention to highly educated children whose parents had immigrated to Britain, often from India.^73^ Concurrent research in America, conducted in the 1980s and 1990s, was assessing how children who were minority ethnic, from a minority culture or spoke English as a second language, as well as those who were poor or female, were less likely to be identified as ‘gifted’ by achievement or IQ testing or teacher recommendations because of the vague definition of ‘giftedness’, misuses of testing and as tests were normed for white, middle-class boys.^74^

Voluntary sector organisations conducted significant work looking to help working-class parents to identify and support their gifted youth. The National Association for Gifted Children, for example, established specialist programmes in ‘priority areas’ of the North East of England, Liverpool, the East End of London and Greater Manchester.^75^ Nonetheless, the organisation also recognised that its membership was disproportionately middle-class and well-educated. In a 1987 survey of 125 member families, only 6 per cent worked in manual professions.^76^ As the survey acknowledged, this did not mean that ‘most gifted children are in the middle classes’, rather, it meant that this organisation had ‘not yet succeeded in attracting as many members from the working classes’. Perhaps, the survey analysis speculated, because of people’s fears of singling out their children or making them appear ‘different’.^77^ These assumptions, Laura Tisdall has argued, were ingrained in the very foundation of Britain’s post-war educational settlement, where the ideals of a ‘healthy child’ and ‘good student’ or ‘citizen’ were modelled around the white, middle-class boy.^78^

These contexts thus frame our thinking about ‘the family’ in 1970s and 1980s debates. The family was a site of great commercial, political, voluntary and professional interest, perceived as able to identify and construct future citizens, and particularly those who could lead a united Europe or restore a flailing British economy. While advertising sought out all parents, and voluntary sector groups sought to analyse their own internal class dynamics, typically, influential factions of media and policy assumed that middle-class families were more likely to have gifted children and to nurture them ‘appropriately’. In turn, middle-class families were more likely to join voluntary organisations in this area and to become activists and spokespeople. This frames the analysis of the rest of this article, which considers how parents and children utilised growing interest in family life to make their own demands and claims visible. This analysis also reiterates how strongly visions of class, race, ethnicity and gender were deeply rooted within the post-war welfare democratic settlement, as feminist critique has demonstrated, and how these systems of bias and discrimination were bolstered in the 1970s and 1980s by perceived crises in productivity, welfare and democracy.^79^ While much existing work on popular individualism focuses on the experiences and critique of ‘ordinary’ or minoritised populations, this article explores how more privileged families also felt neglected in the ageing welfare state, and subsequently utilised their relevant professional and cultural capital to make claims framed around experiential and emotional expertise.^80^

## Rejecting Parental Responsibility

Having been empowered – even expected – to lead on the identification and nurture of gifted children, many parents utilised cultural attention around this phenomenon to highlight the daily challenges of living with a gifted offspring, rather than their future potential. From 1965 to 1970, Harold Wilson’s Labour government legislated for a shift to comprehensive schooling, ending selection at eleven; a policy which, Peter Mandler has shown, reflected longer-standing demands from working- and middle-class parents alike.^81^ The public statements of gifted campaigners in the 1970s and 1980s may initially be read as a part of this story: here, perhaps, primarily middle-class parents deployed the abstract yet popular new language of ‘giftedness’ to seek out educational advantage amidst levelling policy shifts. Notably, however, parents of the gifted did not focus on demanding educational reform. Rather, they wanted to construct new communities of solidarity and sympathy through voluntary action. These parents, as a consequence, framed their discussions around experience and emotion, rather than focusing on discussing policy provision or entitlement.

Across the 1960s to the 1990s, media outlets were interested in representing giftedness in newspapers and on television. Such reporting, particularly in the 1960s, often prioritised professional testimonies. The documentary series *Horizon*, for example, aired a programme called *The Gifted Child* on 30 January 1969, which focused on interviewing teachers, voluntary sector experts and educational psychologists, notably those working at the Yehudi Menuhin School, Surrey, the National Foundation for Educational Research, Brentwood Teachers’ Training College and Westminster School.^82^ Children and parents were also featured in this documentary, but typically being pictured, rather than interviewed, with, for example, visual depictions of children playing instruments under the instruction of teachers and undergoing psychiatric assessment. Nonetheless, despite the focus on professional testimony, this documentary – one of the first to assess this area – featured one brief interview with parents. Furthermore, the edit of this interview which was screened portrayed connections between giftedness and familial disruption, as the mother of a gifted child discussed social stigma and isolation, stating that, when discussing her daughter, ‘one becomes then very aware of who one talks to and of what one says’.^83^ This testimony revealed the co-curation of family discord by parents and television. The mother asserted that she constantly reflected on who she was speaking to, and how they would interpret her words. She was articulate and thoughtful – the type of parent able to guide such media encounters – yet also framed her involvement around emotional struggle.

Families were increasingly a feature of documentaries about giftedness screened in the late 1970s, 1980s and 1990s, and the National Association for Gifted Children gave parents new support to manage, and access to, media engagement. The documentary *In with a Head Start?* (1978) was produced by the Community Programme Unit at the BBC, which looked to create televisual representations of ‘non-professionals, campaigning groups and others’.^84^ *In with a Head Start?* was made in collaboration with the Northern Irish branch of the National Association for Gifted Children. Writing to the organisation’s membership in November 1980 – a sympathetic audience – the group’s chairwoman, Felicity Ehrlich, emphasised the collaborative nature of this production, writing that: ‘every decision was ours, from writing the script to choosing the lettering on titles, from presenting the programme to advising on editing’.^85^ This testimony – and the fact that the National Association subsequently used this documentary as a training video – demonstrates how much the Association gained from this partnership.

Certainly, as a result of this co-production, the final documentary was framed at the beginning and the end by parents’ testimonies.^86^ Furthermore, the National Association and involved parents used this representative space to emphasise the lived challenges of raising gifted youth – as well as the role of the voluntary sector in ameliorating this. Introducing the programme, Ehrlich stated that while people often assumed it must be ‘wonderful’ to have a gifted child, ‘the trouble is that such a child often needs an exceptionally talented mother and father’.^87^ Such parents would typically be exhausted by the physical and mental energies of these children, and left ‘on duty sixteen hours a day’.^88^ Interviewed parents offered similar accounts, describing their children as ‘absolutely exhausting’ and ‘a problem child’– the latter a term concurrently applied to children engaged in crime.^89^ Parents also pushed back on the idea that they could inculcate a sense of national duty within their children, stating that, rather, parenting frequently left them wondering ‘what exactly I was doing wrong’ and unable to keep their child ‘amused’.^90^ These themes were reiterated in parental letters and testimonies published by 1970s and 1980s tabloid newspapers, where parents stated that gifted children could ask questions which exceeded their own knowledge, leaving them ‘embarrassed’ and ‘bewildered’, and also that extended family and friendship groups might be ‘incensed’ by their child’s relative progress, causing broader forms of social rupture.^91^ By the 1997 BBC programme *Challenging Children: A Gifted Child*, documentaries had begun to take close case studies of individual families alone. In this programme, parents of a gifted daughter emphasised that they rarely spent time together and had struggled to access professional support, with one doctor, for example, telling them that their daughter would soon ‘forget’ her giftedness.^92^

These testimonies were powerful because of the broader cultural expectations that emotion and experience be performed and made visible in this period. Parents were able to powerfully mobilise these expectations to raise awareness of giftedness. In doing so, private accounts suggest, these parents hoped to construct new, highly localised, communities of sympathy, support and solidarity. One unsolicited contribution on this topic, for example, from a Mass Observation diarist, was written in September 1984.^93^ In this, the mother-of-two wrote that she had joined the National Association for Gifted Children out of ‘fears for our sons [sic] future happiness’. She described her son as ‘at times quite difficult to cope with’: he had ‘rarely smiled’ as a baby, would issue ‘screams of frustration when his body could not obey his mind’ and he was always easily ‘frightened’ and ‘hysterical’. Speaking for herself and her husband, the mother described feeling ‘frustrated’ and ‘angry’. She joined the National Association not to demand more provision from the welfare state but to find others to speak with, and to seek out help from other parents in similar situations. The mother felt she needed such a specialised community: she had previously tried to speak with other parents in her area or through school but had received a hostile reception, and now felt full of ‘fear’ about sharing her experiences.^94^ The parents of gifted children, then, typically framed their participation in new voluntary cultures around ideas of desperation and struggle rather than entitlement, and they called for solidarity and sympathy more often than change.

The extent to which parents’ narratives about giftedness were disseminated is difficult to gauge. Writing to impress and engage an audience of affected parents and interested professionals, Ehrlich argued that working on the *Open Space* documentary had been ‘well worth the effort’, particularly as the ‘public response was overwhelming’.^95^ What is certainly significant, however, is that these co-constructed voluntary, media and parental portrayals subverted concurrent visions of gifted children as future assets – whether of productivity, democracy or peace. Drawing on their own authority from experience, parents emphasised that children caused lived disruption to everyday life in the present. These representations were mediated by concurrent press and televisual interest in exploring intimate family lives, and by activist interest in bolstering the role of the voluntary sector in offering solutions. Nonetheless, the co-constructed representations which emerged disrupted representations of parents as in a deferential relationship of servitude to both their children and to ‘the state’, responsible for identifying and nurturing their children such that their talents would enrich national futures.

## Disruptive Children’s Accounts

Children also sought to reject the expectations put on them as agents of democracy, peace and productivity – though child-written publications from voluntary groups also show the power of these visions in children’s minds. Indeed, materials published by the National Association provide insight into the ways that education, parents and the voluntary sector placed expectations and duties on gifted children, and the aspects of these duties which children sought to accept or reject. Notably, while parents sought out solidarity and sympathy, young people demanded change. In Autumn 1979, the National Association published an edition of its *Journal of the Gifted Child*, intended for interested professionals and parents. Alongside articles from teachers and educationalists describing their research, the journal also featured an article called ‘Saturday Club’, describing a leisure group organised with the Inner London Education Authority, providing specialist activities for Association members designed to stretch them beyond the school curriculum alone. The article featured a description of this group, a series of pictures of attending children, and two short testimonies: the first an eighty-one-word comment from a ten-year-old boy, the second a 396-word comment from a thirteen-year-old boy – selections which mirrored the disproportionate identification of boys as ‘gifted’. These testimonies were solicited and framed by the National Association, and indeed emphasised the benefits which this group had brought the children’s personal development and family lives. The thirteen-year-old boy reported that the Saturday Club had enabled him to transit from feeling lost at school to socialising with new peers, and had furnished him with new interests in philosophy and photography which, in turn, helped him to find ‘school work more interesting’.^96^

However, these testimonies also demonstrated the influence of national narratives of duty. In his quote, the thirteen-year-old boy stated concern that he could ‘offer nothing of great importance to the world around me’, given that he had ‘no power, no money, no influence and little experience’. Nonetheless, he also emphasised that he felt compelled to ‘reach the end of the road’ only when he had ‘played my part in the world around me’.^97^ The boy thus framed his life purpose in terms of playing ‘a part’ in ‘the world’, and emphasised that he found this daunting, and potentially unrealistic given his structural lack of power. The ten-year-old boy likewise reflected on his social and economic roles. Positioning these as concomitant to his own interests, the boy stated that while his school peers liked to play catch, he liked to ‘talk about the world’ and to discuss ‘the wealth of Great Britain, the world monetary system, and political parties and what they stand for’.^98^ By joining the Saturday Club, the ten-year-old had found older peers who shared these interests. Significantly, therefore, these co-constructed testimonies suggested that national agendas and cultural discourses around gifted children perforated and shaped discussions in the voluntary sector and of children themselves; these grand and abstract visions reframed the daily, social, voluntary and inner lives of gifted youth.

National Association publications also featured gifted children directly resisting ideas of their national role and critiquing existing systems of education and welfare. These challenges recurred frequently in Association magazines published for gifted members, which contained child-authored drawings, stories, book reviews, poetry and letters, and were banded for different age groups. In the Easter 1982 edition of *Gallimaufry*, for example, the magazine aimed at children aged seven to twelve ‘or thereabouts’, one anonymous child provided ‘the story of Ogg’: ‘a normal person with a funny name and a funny looking face’. Ogg – like the children reading this story, and its author – had been identified as of high intelligence. Yet, Ogg was teased by ‘two normal brothers’, and his school would not teach him, fearing that his intelligence would ‘discourage the other boys’. This isolation from familial and educational communities left Ogg living on ‘dole money’ with the one friend who accepted him, despite the fact that, the child author wrote, Ogg ‘could have been a bank teller, an accountant, or even a politician’. Nonetheless, the child concluded, Ogg ‘did not waste his talents – the community wasted them for him’.^99^ This thinly-veiled morality tale therefore satirised and critiqued the ideas that gifted children should not ‘waste’ their talents, and the idea that they should fulfil ‘useful’ positions in politics and the economy. The child author presented damning reflection on these aims, arguing that they were blocked not by the capacity of children themselves, but rather by the inadequacies and discrimination of the state education system, and the social stigma children faced from families and peers. Rather than fulfilling high-flying roles, the author emphasised, gifted children may be left reliant on state-benefits – a significant threat in a 1980s moment where unemployment was stigmatised by conservative policy and press.^100^

These themes and critiques were echoed through other child-centred magazines produced by the National Association. A sixteen-year-old contributor to the Easter 1983 edition of *Apotelesmatics* magazine, for example, wrote a poem about a boy who, despite his high intelligence, could not find work, because: ‘They thought he was mental’.^101^ This poem again emphasised the structural and social barriers which gifted youth faced, placing the burden of blame broadly – on ‘They’. In Autumn 1979, the Association newsletter *Explorers Unlimited* – directed at those who had attended summer camps – published four letters from gifted youth aged twelve to fourteen. These criticised the ‘unfair’ and ‘restrictive’ provisions of the state education system, notably its lack of ‘proper streaming’, teachers who were ‘prejudiced’ and the bullying which came as a result of being ‘different’.^102^ Again, therefore, children, like their parents, were able to use the spaces newly available to them to reframe discussions about giftedness. Instead of focusing on the contribution which gifted children could make to society, these children criticised the education system and the attitudes of their peer groups. The adult editor of this magazine expressed willingness to seek out further views on this, calling for ‘comments on these comments please?’ from child readers, and emphasising that education in particular was a theme ‘that very much concerns each and every Explorer’.^103^ Further publications from the Association’s magazines contained contributions from children who, from lived experience, challenged psychological categorisations of measurement and IQ and emphasised feelings of despair and struggle.^104^

Through the voluntary sector, gifted youth were hence given new opportunities to challenge adult expectations of their role, in part because of the adult vision of these youth as particularly critical and disruptive. The critique of child members of the National Association was broader, more ambitious and more directly targeted than that of their parents. In particular, gifted young people often felt let down by post-war education, despite the post-war interest in ‘ability’ (which, Laura Tisdall has argued, also brought the dangerous assumption that less academic children ‘would never develop into fully mature beings’).^105^ The critique of the gifted young did not typically lead to change, however. Indeed, it was often offered and received somewhat playfully. Many adults represented gifted children’s commentaries as entertainment: a framing visible, for example, in tabloid coverage of children who dismissed psychological testing as ‘silly questions’.^106^ Children, furthermore, often likely enjoyed the performative engagement with adults in press and voluntary spaces, relishing their unique access to adult attention. Overall, looking closely at children’s testimonies demonstrates the significant need to assess relationships and structures that exist within ‘the family’. While difficult to identify, available archival traces also demonstrate the lived impact of welfare and democratic systems on children’s lives, and present children as active participants in calling for national and transnational change.

## Conclusion: What Family Values?

Families – and particularly parents – in this case study were perceived as a key bastion for implementing national agendas by policy makers across Europe in the 1970s and 1980s: responsible for identifying gifted children and for providing an ‘appropriate’ home environment. For a circle of activists and researchers in Britain and continental Europe, parents’ responsibility was to nurture the gifted child in the service of European – or even global – peace and democracy. In Britain, debates led by conservative press and policy makers constructed the gifted child as an asset for pursuing national economic advantage, amidst a perception of ‘crisis’ for post-war welfare institutions and spending. This vision cross-cut Western European alliances, as tabloids expressed interest in how authoritarian states across the globe utilised centralised systems to inculcate nationalist and populist visions in youth populations. Clearly, debates around giftedness were inflected by cultural anxieties about Britain’s place in the world and in Europe, fuelled by the ongoing decline of empire and contested membership of the European Economic Community. Looking at giftedness is hence a powerful proxy through which to understand anxieties about the potential of youth more broadly, and to see how debates around child development, parental responsibility and the boundaries of state power were tied to broader agendas around productivity, democracy and welfare.

Parents shouldered the responsibility for enabling their children to meet these diverse goals – an expectation pushed through media representation, policy rhetoric and educational and psychological practice, as well as in the thriving industry of advertising. Yet, the key message of this article is that families – parents and children alike – were far from passive in accepting such assumptions, but rather renegotiated the meanings of giftedness through activism and everyday life. Often through the National Association, parents used media interest in their lives to portray the challenges of raising a gifted child, in terms of requiring extra time, effort and resources and testing parents’ relationships with peers and family. Parental testimony was framed by media and cultural narratives but nonetheless played a subversive role in redirecting cultural focus from debates about the future purpose and value of the gifted child towards consideration of their lived experience and potentially damaging effects.

Children at times enjoyed voluntary activities open to them because of their label, ‘gifted’. Nonetheless, children also used these voluntary spaces of leisure, and the newsletters and magazines constructed by the National Association, to criticise systems of education and psychology, and to define and debate the challenges of forming peer networks while living with high intelligence. Children’s narratives were sought out by voluntary leaders. The perception of these youth as especially critical, while framing published accounts, also furnished children with unique spaces in which to co-construct representations of their inner worlds and concerns. Looking at these accounts makes clear that many gifted children engaged with national debates about their purpose, and reflected seriously on this, while also constructing alternative narratives around the prioritisation of individual or familial fulfilment.

It is difficult to trace archival evidence of children’s own activism and agency and of the everyday practices of families. Nonetheless, methodologically, this article has demonstrated that archival traces are available, when we look broadly at the range of materials which parents and children were able to create and disseminate. We should take seriously, for example, published poetry, letters and interviews in newspapers and documentaries, as items which were co-produced between children, parents and voluntary organisations and media. We cannot write off these sources; to do so is to assume that families *could not* have held power within systems of knowledge-production and exchange. Rather, however, the children of affluent, eloquent, middle-class families were disproportionately identified as ‘gifted’, and themselves and their parents held multiple forms of cultural and professional capital – as well as experiential expertise – which enabled them to drive media and voluntary narratives, as well as to reflect them. Placing these sources alongside expert-led professional accounts enables us to break down thinking about ‘expertise’ within decades where professional credentials were newly challenged by media and populist politics, and where experiential expertise became increasingly visible as a motivator and justification for voluntary action. In this case, this archival method has demonstrated that families were active agents in negotiating any national quest for welfare, democracy and productivity, and in reshaping the course of these aims and trajectories through activism and daily life. The voluntary sector supported and shared various national agendas around the role of the gifted, but also provided a refuge and series of mechanisms through which parents and children could meet one another, form new virtual and physical communities and subvert national expectations through leisure and political pursuits.

Bringing these sources together brings new perspectives on national and family lives alike. Families have not only ‘fortified the boundaries within which they lived’ through their economic and social behaviours, but they have also challenged and co-constructed them.^107^ In this case study families were, first, participants in the flourishing of new voluntary cultures in 1970s and 1980s Britain, whereby declining previous forms of association, such as trade unions and churches, were supplanted by new social movements, new charities and new campaign groups around a diffuse range of areas, including giftedness. Families were, secondly, drivers of shifts towards Britain as a more ‘individualist’ society in the 1970s and 1980s, as parents and children sought to reject professional and state intervention around giftedness, or a sense of a national purpose, and rather to prioritise their immediate sense of wellbeing and happiness. We may wish to situate this rising sense of individualism amidst new challenges to the post-war settlement made by privatisation and a growing sense of ‘crisis’ in economics and fringe politics. Yet, as Lawrence has argued, this rise of individualism did not necessarily reflect the decline of ‘community’, welfare, nor, necessarily, a ‘crisis’.^108^ Rather, the rise of ‘individualism’ merely meant that families began to reconfigure their immediate and social relationships actively, in terms of choice and affection, rather than by proximity or need – in this case study, families of gifted youth forged local and transnational bonds with other affected families, motivating their activism and changing their daily routines. The families in this article, notably, chose to make their experiences and emotions of struggle central to their activism: we must pay attention and carefully analyse why parents found it more easy, powerful and necessary to articulate their feelings in the distinct contexts of the 1970s and 1980s.

New Right rhetoric and facets of psychiatry and popular culture sought to position families as vulnerable in the 1970s and 1980s, highlighting family violence, poor systems of education and the decline of two-parent households. Yet, looking at sources produced by parents and children instead suggests that privileged families were increasingly ‘strong’, able to access and utilise new opportunities in the media and voluntary sector and to reshape debates in education and psychology through descriptions of their lived experience. The empowerment of these families raised its own problems: the value placed on experiential expertise, and the ability of highly educated families to negotiate media networks, left little focus on how ‘representative’ these families were, nor on how the identification of ‘gifted’ youth replicated historic structures of bias and discrimination. ‘[S]elf-appointed members of the articulate middle-class’ were able to utilise media and policy interest to promote the ‘development’ of their own children.^109^ This familial activism shifted national and transnational debate, but did not fundamentally critique, nor seek to overthrow, the biases built in to underlying systems of welfare and democracy.

## Footnotes

1 ‘Is Teaching the Best Judge of a Gifted Child?’, *Daily Telegraph*, 9 Sep. 1977, 17.

2 Ibid.

3 Ibid.

4 Ibid.

5 Ibid.

6 This research was particularly prominent in the United States. See for example: E. Susanne Richert, ‘Identification of Gifted Children in the United States: The Need for Pluralistic Assessment’, *Roeper Review*, 8, 2 (1985), 68–72; E. Susanne Richert, ‘Rampant Problems and Promising Practices in the Identification of Disadvantaged Gifted Students’, *Gifted Child Quarterly*, 31, 4 (1987), 149–54; Donna Y. Ford, ‘The Underrepresentation of Minority Students in Gifted Education: Problems and Promises in Recruitment and Retention’, *The Journal of Special Education*, 32, 1 (1998), 4–14; J. John Harris and Donna Y. Ford, ‘Hope Deferred Again: Minority Students Underrepresented in Gifted Programs’, *Education and Urban Society*, 31, 2 (1999), 225–37. Important recent research has discussed how these same tests led to Black children being unfairly identified as ‘subnormal’, often limiting their educational opportunities and damaging their self-esteem: Rob Waters, *Thinking Black: Britain, 1964*–*1985* (Berkeley: University of California Press, 2018); see also Steve McQueen’s *Small Axe* episode on education (2020) and the BBC’s documentary *Subnormal* (2021).

7 Siân Pooley and Kaveri Qureshi, ‘Introduction’, in Siân Pooley and Kaveri Qureshi, eds., *Parenthood Between Generations: Transforming Reproductive Cultures* (Oxford: Berghahn Books, 2016), 20; Adrian Bingham, *Family Newspapers?: Sex, Private Life, and the British Popular Press, 1918*–*1978* (Oxford: Oxford University Press, 2009), 2, 10; Angela Davis, *Modern Motherhood: Women and Family in England, 1945*–*2000* (Manchester: Manchester University Press), Chapter 5, ‘“Experts” and Childcare “Bibles”: Mothers and Advice Literature’.

8 Materials in the archives of the National Association for Gifted Children provide accounts of the organisation’s inception and early work. See for example: National Association for Gifted Children archives, Bletchley (hereafter NAGC), National Association for Gifted Children Flyer, 2, undated but likely between 1965 and 1975 given names of director and secretary listed; NAGC, *Looking to Their Future: The News Letter of the National Association for Gifted Children*, June 1976; Denis W. Ockleton, ‘Margaret Branch’, 1.

9 Elaine Tyler May, *Homeward Bound: American Families in the Cold War Era* (New York: Basic Books, 1988), 10.

10 Lynn M. Thomas, ‘Historicising Agency’, *Gender & History*, 28, 2 (2016), 335.

11 Chris Millard, ‘Using Personal Experience in the Academic Medical Humanities: A Genealogy’, *Social Theory & Health*, 18 (2019), 184–98; Mona Gleason, ‘Avoiding the Agency Trap: Caveats for Historians of Children, Youth, and Education’, *History of Education*, 45, 4 (2016), 446–59; Thomas, ‘Historicising Agency’, 324–39; Susan A. Miller, ‘Assent as Agency in the Early Years of the Children of the American Revolution’, *Journal of the History of Childhood and Youth*, 9, 1 (2016), 48–65; Tatek Abebe, ‘Reconceptualising Children’s Agency as Continuum and Interdependence’, *Social Sciences*, 8, 3 (2019), 1–16; Kathryn Gleadle and Ryan Hanley, ‘Children Against Slavery: Juvenile Agency and the Sugar Boycotts in Britain’, *Transactions of the Royal Historical Society*, 30 (2020), 97–117.

12 Emily Robinson, Camilla Schofield, Florence Sutcliffe-Braithwaite and Natalie Thomlinson, ‘Telling Stories about Post-war Britain: Popular Individualism and the “Crisis” of the 1970s’, *Twentieth Century British History*, 28, 2 (2017), 268–304.

13 Jon Lawrence, *Me, Me, Me? The Search for Community in Post-war England* (Oxford: Oxford University Press, 2019).

14 See the critique of anti-psychiatry in this period, for example in: David Cooper, *The Death of the Family* (London: Penguin, 1971); R.D. Laing and Aaron Esterson, *Sanity, Madness and the Family* (London: Penguin, 1973).

15 For key context on this – and on how these debates related to those around autism and psychosis – see Bonnie Evans, ‘Between Instincts and Intelligence: The Precarious Sciences of Child Identity in Twentieth-Century Britain’, *Psychoanalysis and History*, 21, 2 (2019), 171–92.

16 For longer histories of early debates around IQ see: Serge Nicholas, Bernard Andrieu, Jean-Claude Croizet, Rasyid B. Sanitioso and Jeremy Trevelyan Burman, ‘Sick? Or Slow? On the Origins of Intelligence as a Psychological Object’, *Intelligence*, 41, 5 (2013), 699–711; Gillian Sutherland, ‘The Magic of Measurement: Mental Testing and English Education 1900–40’, *Transactions of the Royal Historical Society*, 27 (1977), 135–53; Sally Shuttleworth, *The Mind of the Child: Child Development in Literature, Science and Medicine, 1840*–*1900* (Oxford: Oxford University Press, 2010). In later periods, significant texts around giftedness were written by the Hungarian-Dutch psychologist Géza Révész, who wrote *Psychology of a Musical Prodigy* (1925), and from Dutch psychologist Jan Waterink, who wrote *Giftedness as a Qualification of the Personality* (1938).

17 Nicholas, Andrieu, Croizet, Sanitios and Burman, ‘Sick? Or Slow?’, 701.

18 Mathew Thomson, *The Problem of Mental Deficiency: Eugenics, Democracy and Social Policy in Britain, 1870*–*1959* (Oxford: Oxford University Press, 1998); Mathew Thomson, ‘Mental Hygiene as an International Movement’, in Paul Weindling, ed., *International Health Organisations and Movements, 1918*–*1939* (Cambridge: Cambridge University Press, 1995), 283–304.

19 Julia Barbara Köhne, ‘The Cult of the Genius in Germany and Austria’, in Joyce E. Chaplin and Darrin M. McMahon, *Genealogies of Genius* (Basingstoke: Palgrave, 2016), 116–17, 118, 131.

20 Franz J. Mönks, Michael W. Katzko and Herman W. Van Boxtel, eds., *Education of the Gifted in Europe: Theoretical and Research Issues, Report of the Educational Research Workshop held in Nijmegen (The Netherlands), 23*–*26 July 1991* (Netherlands: Swets & Zeitlinger B.V., 1992), Table of Contents.

21 Herman W. van Boxtel, ‘Final Report’, in Mönks, Katzko and Van Boxtel, *Education of the Gifted in Europe*, 26–7.

22 Ibid., 27.

23 Ibid., 18, 73, back cover.

24 Kurt A. Heller, ‘Giftedness Research and Education of the Gifted and Talented in Germany’, in ibid., 76.

25 ‘Mental resources’ of the gifted child are discussed in ibid., 76. The idea of the child as an ‘agent of future promise’ is discussed by Laura King and Vicky Crewe, for example in: ‘Agents of Future Promise’ project website <https://childrenofthefuture.leeds.ac.uk/about/> (18 July 2019); Laura King, Vicky Crewe and Lindsey Dodd, ‘Children and Notions of “the Future”‘, *History & Policy*, 5 Feb. 2015 <http://www.historyandpolicy.org/opinion-articles/articles/children-and-notions-of-the-future> (18 July 2019).

26 Heller, ‘Giftedness Research and Education’, 76; European Council for High Ability news, 8, 2, 2 Sep. 1994, as cited in ‘The History of ECHA’, European Council for High Ability website, <https://www.echa.info/history-25-years/content/2-about-echa> (18 July 2019).

27 NAGC, ‘International Exchanges & Visits’, *National Association Gossip Column*, Issue 8, Summer Term 1991.

28 NAGC, *NAGC Newsletter*, Summer 1995, ‘GIFT Went Hungarian’, 6.

29 See: Matthias Neumann, ‘Children Diplomacy during the Late Cold War: Samantha Smith’s Visit of the “Evil Empire”‘, *History*, 104, 360 (2019), 277–8.

30 Richard Ivan Jobs, *Backpack Ambassadors: How Youth Travel Integrated Europe* (Chicago: University of Chicago Press, 2017).

31 For further context about British debates around the European Economic Community in the 1970s, please see: Robert Saunders, *Yes to Europe! The 1975 Referendum and Seventies Britain* (Cambridge: Cambridge University Press, 2018).

32 On changing manifestations of this, from Thatcherite policy to the Coalition government of 2010–15, see: Paul Daniel, ‘Conservative Policy and the Family’, in Hugh Bochel, ed, *The Conservative Party and Social Policy* (Bristol: Policy Press, 2011), 197–214. See also: Martin Durham, *Sex and Politics: The Family and Morality in the Thatcher Years* (Basingstoke: Macmillan Education, 1991).

33 On which see: Pat Thane, *Unequal Britain: Equalities in Britain Since 1945* (London: Continuum, 2010); Pat Thane, *Divided Kingdom: A History of Britain, 1900 to the Present* (Cambridge: Cambridge University Press, 2018); Mathew Thomson, *Lost Freedom: The Landscape of the Child and the British Post-War Settlement* (Oxford: Oxford University Press, 2013).

34 For a discussion of this ‘white heat’ moment – which predated these concerns by a debate but were revived in the conservative press – see David Edgerton, ‘The “White Heat” Revisited: The British Government and Technology in the 1960s’, *Twentieth Century British History*, 7, 1 (1996), 53–82. This framing also drew on American framing around the gifted child, which was very powerful in policy and voluntary circles in Britain in this period (on this see L. Jolly, ‘The National Defense Education Act, Current STEMP Initiative, and the Gifted’, *The Gifted Child Today*, 32 (2009), 50–3).

35 Discussing the positioning and role of the *Daily Mail*: Adrian Bingham, *Family Newspapers? Sex, Private Life, and the Popular Press, 1918*–*1978* (Oxford: Oxford University Press, 2009), 5, 11. See, for example, on the above themes: Christopher Rowlands, ‘Why Gifted Claire is a Misfit’, *Daily Mail*, 9 Aug. 1978, 6; Ian Smith, ‘Treated like a Freak for Being Clever’, *Daily Mail*, 28 Nov. 1978, 6–7.

36 K.D. Barritt and E. Price, ‘Come on, Britain, Use Your Brains’, *Daily Mail*, 20 Apr. 1979, 36; June Southworth, ‘Everything We All Need to Know about Gifted Children’, *Daily Mail*, 18 Aug. 1979, 6–7.

37 Smith, ‘Treated like a Freak’, 6–7; Southworth, ‘Everything We All Need to Know’, 6–7; Jill Crompton, ‘Letters’, *Daily Mail*, 15 June 1977, 27.

38 Rowlands, ‘Why Gifted Claire is a Misfit’, 6; Smith, ‘Treated like a Freak’, 6–7.

39 Peter Chambers, ‘What Happens to IQ Kids Who Are Too Bright for the Teachers?’, *Daily Express*, 17 Apr. 1973, 11.

40 Lord Carrington, ‘Needs of Gifted Children’, House of Lords, Hansard, Fifth Series, 14 May 1969, vol. 302, col. 126. Discussion of Lord Carrington’s role as a ‘distinguished sponsor’, and of his work in raising this debate, is here: NAGC, ‘House of Lords Debate’, *Newsletter*, May 1969, 1.

41 Carrington, ‘Needs of Gifted Children’, vol. 302, col. 129.

42 Chambers, ‘What Happens to IQ Kids?’, 11.

43 Southworth, ‘Everything We All Need to Know’, 6–7.

44 Lord Snow, ‘Needs of Gifted Children’, House of Lords, Hansard, Fifth Series, 14 May 1969, vol. 302, col. 162.

45 Quote is from the ‘white heat’ speech, as cited in Glen O’Hara, ‘“The Russian Revolution Has not yet Taken Place”: British Views of the Soviet Economy between the 1950s and the 1970s’, *Historical Research*, online access first 10 Sep. 2019, 18.

46 Snow, ‘Needs of Gifted Children’, vol. 302, col. 164.

47 Henry Collis, ‘At Work in Bulgaria’, *Looking to Their Future*, May 1977, 9–10.

48 Sebastian Coe, ‘The Public’s Responsibility to Promote Excellence’, *European Journal of High Ability*, 1, 1 (1991), 10.

49 Ibid., 10.

50 Conference proceedings in: Bodleian Library, Joy Gibson and Prue Chennells, eds., *Gifted Children: Looking to their Future* (Essex: The Anchor Press, 1976).

51 ‘Is There a Genius in Your Family?’, *Daily Mirror*, 11 Sep. 1975, 7; ‘How to Spot a Gifted Child’, *Daily Express*, 4 Apr. 1991, 37.

52 Patricia Mason and Juliet Essen, *The Social, Educational, and Emotional Needs of Gifted Children* (Cicely Northcote Trust, 1987), 25.

53 British Film Institute, *Open Door: In with a Head Start?*, 7 Dec. 1978.

54 On the development of consumer culture see: Matthew Hilton, ‘Social Activism in an Age of Consumption: The Organized Consumer Movement’, *Social History*, 32, 2 (2007), 121–43; Matthew Hilton, ‘The Death of a Consumer Society’, *Transactions of the Royal Historical Society*, 18 (2008), 211–36.

55 ‘Horlicks’, *The Times*, 14 Nov. 1950, 2; ‘Horlicks’, *Daily Telegraph*, 16 Nov. 1950, 6.

56 Ibid.

57 Ibid.

58 See for example: NAGC, Professor R.S. Illingworth, ‘Predicting Potential and Helping the Child to Achieve It’, *Looking to Their Future*, Nov. 1977, 1–2, in which the professor, looking at how to predict child development, explores ‘the Mothers’ role’ specifically, and offers gendered advice around how women should behave in pregnancy and early childhood. This echoes the professional focus on offering advice literature to mothers, in particular, throughout modern British history, as traced for example by Angela Davis in *Modern Motherhood*, Chapter 5.

59 ‘IQ Pills Cost Firm £86,000’, *Daily Express*, 14 Oct. 1992, 12–13.

60 Advert, *Daily Mail*, 2 Mar. 1991, 23.

61 Ibid.; ‘Multiple Display Advertising Items’, *Daily Mail*, 5 Mar. 1991, 20; Advert, *The Times*, 28 Feb. 1991, 21; Advert, *Guardian*, 28 Feb. 1991, 39; Advert, *Daily Express*, 11 Mar. 1991, 10.

62 Advert, *Daily Mail*, 2 Mar. 1991, 23; ‘Can You REALLY Make Your Kids Brighter?’, *Daily Mirror*, 28 Feb. 1991, 11; ‘The A–E of Your Child’s IQ’, *Daily Express*, 28 Feb. 1991, 27.

63 See on this: Deborah Cohen, *Family Secrets: The Things We Tried to Hide* (London: Penguin, 2014); Pat Thane, ‘Family Life and “Normality” in Postwar British Culture’, in Richard Bessel and Dirk Schumann, eds., *Life After Death Approaches to a Cultural and Social History of Europe during the 1940s and 1950s* (Cambridge: Cambridge University Press, 2003), 193–210; Martin Durham, *Sex and Politics: The Family and Morality in the Thatcher Years* (Basingstoke: Palgrave Macmillan, 1991); Laura King, *Family Men: Fatherhood and Masculinity in Britain, c. 1914*–*1960* (Oxford: Oxford University Press, 2015), 7; Davis, *Modern Motherhood*; Pooley and Qureshi, *Parenthood Between Generations*.

64 On anxieties about professional expertise at this point, and how parents were able to respond to, and utilise, this, see: Jennifer Crane, *Child Protection in England, 1960*–*2000: Expertise, Experience, and Emotion* (London: Palgrave, 2018).

65 Lawrence, *Me, Me, Me?*

66 Lord Aberdare, ‘Needs of Gifted Children’, House of Lords, Hansard, Fifth Series, 14 May 1969, vol. 302, col. 123.

67 Ibid., vol. 302, col. 156.

68 Lord James of Rusholme, ‘Needs of Gifted Children’, House of Lords, Hansard, Fifth Series, 14 May 1969, vol. 302, col. 171; Roger Young, ‘James, Eric John Francis, Baron James of Rusholme’, *Oxford Dictionary of National Biography*, 8 Oct. 2009 <https://www.oxforddnb.com/view/10.1093/ref:odnb/9780198614128.001.0001/odnb-9780198614128-e-51145> (2 Oct. 2019).

69 British Film Institute, 79570, *Horizon: The Gifted Child*, 30 Jan. 1969.

70 Southworth, ‘What Becomes of Child Prodigies?’, 3.

71 Carrington, ‘Needs of Gifted Children’, House of Lords, Hansard, Fifth Series, 14 May 1969, vol. 302, col. 124; ‘Is There a Genius in Your Family?’, *Daily Mirror*, 11 Sep. 1975, 7; Lynda Lee-Potter, ‘Our Son, the New Captain of Eton!’, *Daily Mail*, 26 July 1980, 7.

72 Ibid.

73 In keeping with the most common visual depictions of gifted children in newspapers over this period, this *Daily Mail* article discusses ‘a fair-haired and pretty little girl with a very winning smile’: Rowlands, ‘Why Gifted Claire is a Misfit’, 6. However, tabloids also emphasised the backgrounds of second-generation immigrant gifted children. For example, this *Daily Mail* article describes a gifted child whose parents ‘arrived in Britain 24 years ago from Bangalore, India’: Richard Wallace, ‘Wonder Boy Finds Celebrity – and Other 9-Letter Words’, *Daily Mail*, 25 Apr. 1988, 13.

74 See for example: Richert, ‘Identification of Gifted Children’; Richert, ‘Rampant Problems and Promising Practices’; Ford, ‘The Underrepresentation of Minority Students’; Harris and Ford, ‘Hope Deferred Again’.

75 NAGC, Twelfth Annual General Meeting held at the Royal Institute, 3 Nov. 1979, Director’s Report, 2; NAGC, Twelfth Annual General Meeting held at the Royal Institute, 3 Nov. 1979, Chairman’s Report, 1; NAGC, *Looking to their Future*, Mar. 1979, ‘Gifted Children and Inner City Areas’, 1.

76 Mason and Essen, *Needs of Gifted Children*, 6.

77 Ibid., 3, 6.

78 Laura Tisdall, *A Progressive Education? How Childhood Changed in Mid-Twentieth-Century English and Welsh Schools* (Manchester: Manchester University Press, 2020), particularly Chapters 5 and 6.

79 See for example: Hilary Land, ‘Who Cares for the Family?’, *Journal of Social Policy*, 7, 3 (1978), 257–84; Mary McIntosh, ‘Feminism and Social Policy’, *Critical Social Policy*, 1, 1 (1981), 32–42; Elizabeth Wilson, *Women and the Welfare State* (London: Tavistock Publications, 1977); Jennifer Dale and Peggy Foster, *Feminists and State Welfare* (Abingdon: Routledge, 1986); Janet Finch, *Family Obligations and Social Change* (Oxford: Polity, 1989); Jen Dale, ‘Feminists and the Development of the Welfare State – Some Lessons from Our History’, *Critical Social Policy*, 16 (1986), 57–65. For critical reading of such work see: Sheila Blackburn, ‘How Useful Are Feminist Theories of the Welfare State?’, *Women*’*s History Review*, 4, 3 (1995), 369–94.

80 Robinson, Schofield, Sutcliffe-Braithwaite and Thomlinson, ‘Telling Stories about Post-war Britain’, 268–304; Lawrence, *Me, Me, Me?*

81 Peter Mandler, *The Crisis of the Meritocracy? Britain*’*s Transition to Mass Education Since the Second World War* (Cambridge: Cambridge University Press, 2020), 34–5, 48–9, 54–5, 60–3, 180–205; Peter Mandler, ‘Educating the Nation I: Schools’, *Transactions of the Royal Historical Society*, 24 (2014), 5–28.

82 British Film Institute, 79570, *Horizon: The Gifted Child*, 30 Jan. 1969.

83 Ibid.

84 Patricia Holland and Ieuan Franklin, ‘Editorial Introduction: Opening Doors: the BBC’s Community Programme Unit 1973–2002’, *History Workshop Journal*, 82, 1 (2016), 213–34.

85 NAGC, ‘Goings on in Northern Ireland, *Looking to their Future*, Nov. 1980, 13.

86 To read about the work of the Community Programme Unit – that worked with community groups to put their experiences on to television – see: Giles Oakley with Peter Lee-Wright, ‘Opening Doors: The BBC’s Community Programme Unit 1973–2002’, *History Workshop Journal*, 82, 1 (2016), 213–34.

87 British Film Institute, *Open Door: In with a Head Start?*, 7 Dec. 1978.

88 Ibid.

89 Ibid.

90 Ibid.

91 Paula James, ‘The Loneliness of a Bright Child’, *Daily Mirror*, 11 Apr. 1972, 9; Smith, ‘Treated like a Freak’; Geoffrey Levy, ‘Watch Out, Genius at Work – and One’s Born Every Week’, *Daily Express*, 20 Aug. 1981, 9; Mrs Grey, ‘Family Affair’, *Daily Express*, 23 Jun. 1981, 24.

92 British Film Institute, 492504, QED, *Challenging Children: A Gifted Child*, 25 Sep. 1997.

93 The Keep, Mass Observation archives, G848, ‘Membership of the National Association for Gifted Children’, 18 Sep. 1984.

94 Ibid.

95 NAGC, ‘Goings on in Northern Ireland’, *Looking to Their Future*, Nov. 1980, 13.

96 Bodleian Library, Per 264505 e. 4, ‘National Association for Gifted Children’, *Journal of the Gifted Child*, Autumn 1979, ‘Saturday Club’, 46.

97 Ibid., 50–1.

98 Ibid., 46.

99 NAGC, *Gallimaufry* magazine, Easter 1982, ‘This is the Story of Ogg’.

100 See on this: Stuart Ball, *Dole Queues and Demons: British Election Posters from the Conservative Party Archive* (Oxford: Bodleian Library, 2011), and on the broader context of stigma against maternity benefits at this time, specifically, Pat Thane and Tanya Evans, *Sinners? Scroungers? Saints?: Unmarried Motherhood in Twentieth Century England* (Oxford: Oxford University Press, 2012), Chapter 8: ‘The Struggle Continues: 1980s–1990s’.

101 NAGC, *Apotelesmatics* magazine, Easter 1983, ‘Jim’.

102 NAGC, *Explorers Unlimited* magazine, Autumn 1979 bulletin, ‘Education Forum’, 1–2.

103 Ibid., 2.

104 NAGC, *Questors Ho!* magazine, 1985, ‘Measurement’, 9; NAGC, *Questors Ho!* magazine, 1985, ‘The Orphan’s Loneliness’, 9; National Association for Gifted Children archives, Bletchley, *Dialogue* magazine, Easter 1982, ‘Someone’s Shoes when They Die’; NAGC, *Questors Ho!* magazine, undated, ‘The Dead’, 5.

105 Tisdall, *A Progressive Education?*, 6, 15, 122, 248.

106 ‘Two in Every 100 – the Children Who Behave like Adults’, *Daily Express*, 25 Mar. 1971, 6.

107 Tyler May, *Homeward Bound*, 13.

108 Lawrence, *Me, Me, Me?*

109 ‘Is Teaching the Best Judge of a Gifted Child?’, 17.

